# Assessing Larval Zebrafish Survival and Gene Expression Following Sodium Butyrate Exposure and Subsequent Lethal Bacterial Lipopolysaccharide (LPS) Endotoxin Challenge

**DOI:** 10.3390/toxins15100588

**Published:** 2023-09-23

**Authors:** Mary X. Wang, Umesh K. Shandilya, Xiang Wu, David Huyben, Niel A. Karrow

**Affiliations:** Department of Animal Biosciences, University of Guelph, Guelph, ON N1G 2W1, Canada; xwang37@uoguelph.ca (M.X.W.); ushand@uoguelph.ca (U.K.S.); xwu13@uoguelph.ca (X.W.); huybend@uoguelph.ca (D.H.)

**Keywords:** *Danio rerio*, sodium butyrate, *Pseudomonas aeruginosa*, lipopolysaccharide

## Abstract

As aquaculture production continues to grow, producers are looking for more sustainable methods to promote growth and increase fish health and survival. Butyrate is a short-chain fatty acid (SCFA) with considerable benefits to gut health, and in recent years, butyrate has been commonly used as an alternative to antimicrobials in livestock production. In this study, we aimed to assess the protective effects of sodium butyrate (NaB) on larval zebrafish subjected to a lethal *Pseudomonas aeruginosa* lipopolysaccharide (LPS) endotoxin challenge and to elucidate potential protective mechanisms of action. Larval zebrafish were pre-treated with 0, 3000, or 6000 μM NaB for 24 h at 72 h post-fertilization (hpf), then immune challenged for 24 h with 60 μg/mL of LPS at 96 hpf. Our results demonstrate that larval zebrafish pre-treated with 6000 μM of NaB prior to lethal LPS challenge experienced significantly increased survival by 40%, and this same level of NaB significantly down-regulated the expression of pro-inflammatory *Tumor Necrosis Factor α* (*TNF-alpha*). Findings from this study are consistent with the beneficial effects of NaB on other vertebrate species and support the potential use of NaB in aquaculture.

## 1. Introduction

Aquaculture production has increased over the past few decades, with production of aquatic animals increasing by 60% in 2020 when compared to the 1990s [1]. However, there are substantial growth and mortality losses due to microbial infections and stressors related to production management and climate change [1]. In addition, antimicrobials can no longer be relied on to prevent or treat microbial infections due to increasing concerns over antimicrobial resistance. Identifying sustainable ways to reduce fish losses and increase disease resistance as well as production is vital in maintaining global food security and supply chains.

Short-chain fatty acids (SCFA) are the end products of bacterial fermentation of dietary fiber in the gut. The most common SCFAs are acetate, propionate, and butyrate, and they have all been shown to elicit beneficial effects in many species, including humans, livestock, and even some fish species [2,3]. In mammalian species, butyrate has been shown to aid in gut epithelial barrier integrity and to elicit immunomodulatory effects [4,5,6], thereby resulting in greater overall nutrient absorption and weight gain and resistance to microbial infection. While the beneficial effects of butyrate on the performance in mammals are well documented, there is much to be learned with regard to potential benefits to aquaculture species.

Zebrafish (*Danio rerio*) are small freshwater fish whose popularity as a vertebrate model has increased in recent decades due to their high fecundity, ease of maintenance, and transparency during their early stages of life [7]. Moreover, zebrafish have a very well-characterized genome, and the acquired immune system of zebrafish is not functional until 3–6 weeks of age [8]. Collectively, these attributes make larval zebrafish an ideal model for studying inflammation and related immunopathology, and it stands to reason that zebrafish may also be a suitable model for cultured aquaculture species.

Diseases caused by bacterial pathogens pose a severe risk to cultured fish production [1]. Several types of Gram-negative bacteria, such as *Aeromonas* spp., *Flavobacterium* spp. And *Pseudomonas* spp. are a threat to cultured fishes [9,10], and more bacterial species are expected to emerge in fish as water ecosystems are increasingly affected by climate change [11]. *P. aeruginosa* is found in several ecological environments, including water and soils [12], and is an opportunistic fish pathogen responsible for causing *Pseudomonas* septicemia in stressed fish that results in high mortalities, desquamation of scales and skin ulcerations [13,14]. *P. aeruginosa* can survive under various climates and conditions and is a common source of infection globally [15,16]. Lipopolysaccharide (LPS) endotoxin is a major virulence factor making up the extracellular membrane of Gram-negative bacteria [17], including *P. aeruginosa*, and is responsible for eliciting immunopathological inflammation at high concentrations. Therefore, *P. aeruginosa* LPS was chosen in this study as an endotoxin model of bacterial infection. 

**Figure 1:**
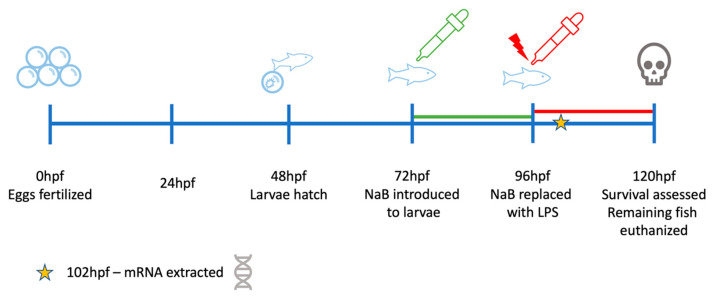
Experimental timeline illustrating NaB exposure to larval zebrafish at 72 h post-fertilization (hfp) followed by immune challenge at 96 hpf with *P. aeruginosa* lipopolysaccharide (LPS) for 24 h.

This study aimed to assess the beneficial effects of sodium butyrate (NaB) on larval zebrafish during a lethal immune challenge with *P. aeruginosa* LPS (Figure 1). The expression of candidate genes coding LPS pattern-recognition receptors, cytokines, signaling molecules, and adhesion molecules was assessed to interrogate potential protective pathways in fish. It was hypothesized that NaB would offer a survival advantage during a lethal immune challenge with LPS in a concentration-dependent manner due to its presumed anti-inflammatory properties and that gene expression related to immune function would be modulated by NaB. 

## 2. Results

### 2.1. Survival Following LPS Immune Challenge

**Figure 2:**
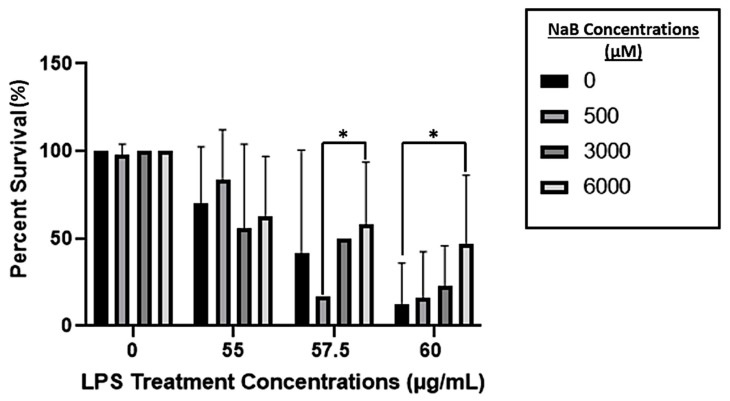
Percent survival of larval zebrafish pretreated with NaB between 72–96 h post-fertilization (hpf), then lethal immune challenged with *P. aeruginosa* lipopolysaccharide (LPS) for 24 h. Each bar represents 18 to 78 fish, as this graph represents 3 independent exposure trials as well as 6 exposure trials that were run in parallel with the gene expression phase of the study. Data are presented as means plus standard error bars, and significant differences are denoted by (*) *p* < 0.01.

Survival of treatment groups exposed to LPS was significantly different from the survival of treatment groups exposed to NaB + LPS in a concentration-dependent manner (*p* < 0.001). Exposure to 6000 μM NaB for 24-h prior to an immune challenge with 60 μg/mL of LPS significantly improved larval zebrafish survival when compared to the control treatment group exposed to 0 μM NaB and challenged with 60 μg/mL LPS (*p* = 0.012; Figure 2).

### 2.2. qPCR Analysis

**Figure 3:**
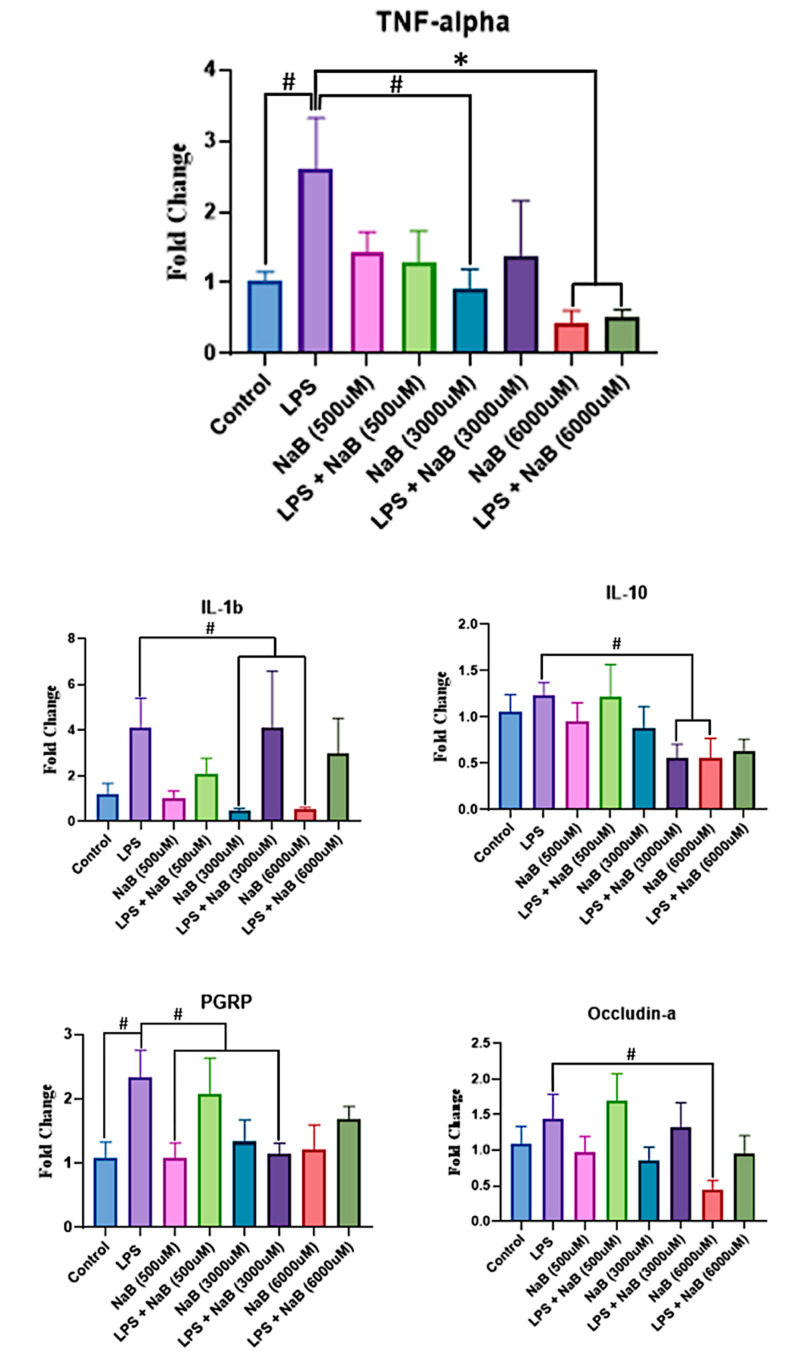
Relative differences in gene expression of larval zebrafish pretreated with NaB between 72–96 h post-fertilization (hpf), then immune challenged for 6 h with *P. aeruginosa* lipopolysaccharide (LPS). Data are presented as means plus standard error bars, and (*) denotes significance (*p* < 0.01), whereas (#) denotes a trend (*p* < 0.1) relative to the positive LPS control treatment. Notes: *TNF-alpha*, Tumor necrosis factor α; *IL-1β*, Interleukin 1β; *IL-10*, Interleukin 10; *PGRP*, Peptidoglycan recognition protein.

The noteworthy changes in gene expression based on treatment are summarized in Figure 3. LPS alone induced *TNF-alpha* expression when compared to the EW control group (*p* = 0.093). Also, exposure to 6000 μM of NaB alone significantly reduced *TNF-alpha* expression when compared to the LPS positive control group (*p* = 0.013). Moreover, exposure to 6000 μM of NaB followed by a subsequent LPS challenge also significantly reduced *TNF-alpha* expression when compared to the LPS control group (*p* = 0.017).

The expression of all other candidate genes was not significantly different based on treatment; however, some trends (*p* < 0.1) were observed. Exposure to 60 μg/mL LPS alone resulted in higher *PGRP* levels than in the EW control group (*p* = 0.073) and in the 500 μM NaB treatment group (*p* = 0.071). Pre-treatment with 3000 μM NaB followed by immune challenge with LPS tended to attenuate *PGRP* expression levels (*p* = 0.093). With respect to *occludin-a*, exposure to 60 μg/mL LPS did not significantly induce expression levels when compared to the EW control; however, when compared to the treatment group exposed to 6000 μM NaB alone, the LPS treatment tended to have higher *occludin-a* levels (*p* = 0.076). For *IL-10*, while exposure to LPS did not significantly induce expression levels when compared to the EW control group, higher *IL-10* levels were observed in the treatment groups exposed to both 3000 μM NaB + 60 μg/mL LPS (*p* = 0.093) and 6000 μM NaB alone (*p* = 0.094). Lastly, while exposure to LPS did not significantly induce *IL-1β* levels when compared to the EW control group, *IL-1β* levels in the LPS treatment tended to be higher than levels measured in the 3000 μM NaB (*p* = 0.060) and 6000 μM NaB treatment groups (*p* = 0.062).

## 3. Discussion

The beneficial effects of NaB on growth performance in finfish species have been explored in recent years [3,18], while the effect NaB has on survivability remains poorly documented. Currently, the known benefits of NaB across species mainly pertain to improved gut health and its ability to act as a histone deacetylase inhibitor (HDACi), thereby giving rise to its immunomodulatory abilities [19], as HDACis are known to affect the expression of *IL-10*, *IL-1β*, and *TNF-alpha* [20,21]. Improved growth performance can be mainly attributed to the improved gut health imparted by NaB, including healthier colonocytes and stronger tight junctions that result in decreased translocation of harmful materials from the gut to the bloodstream [5]. However, the HDACi-mediated immunomodulatory abilities of NaB demonstrate its potential to increase survivability during times of bacterial infection and/or immunopathological inflammation [22]. The results of this study demonstrate that, while NaB alone does not result in different survival rates, in the presence of a bacterial stressor like LPS, prior aqueous exposure to NaB results in a significant concentration-dependent increase in survival rate. Exposure to 6000 μM NaB increased survival of larval zebrafish immune challenged with 60 μg/mL LPS from ~10% to ~50%, resulting in a 40% relative survival increase.

As an HDACi, one of the known immunomodulatory effects of NaB in other species is the down-regulation of *TNF-alpha*, contributing to its anti-inflammatory properties [23]. In humans, butyrate promotes the differentiation of pro-inflammatory M1 macrophages, which are stimulated by LPS treatment to an M2 phenotype that promotes tissue repair, in part by down-regulating the expression of *TNF-alpha* [24]. Contrarily, in sea bass, supplementation of butyrate in the diet is reported to increase the expression of *TNF-alpha* in the intestines and promote the turnover of cells within the intestinal crypts [25]. This variation in the expression of *TNF-alpha* in response to butyrate could be due to the fact that butyrate is not known to be naturally occurring within the zebrafish gut [26]. Moreover, LPS was not used to elicit an inflammatory response within the sea bass but rather a soybean meal diet [25]. It is important to note that the ultimate effect of butyrate remained the same between humans and sea bass: both promote tissue repair and/or cell turnover [25]. Nonetheless, the present study demonstrated that NaB significantly reduced the overall expression of *TNF-alpha* in a concentration-dependent manner, compared to *TNF-alpha* expression in response to LPS alone, and was able to significantly improve survival during an LPS immune challenge. Whether this increase in survival during an LPS immune challenge is directly linked to *TNF-alpha* expression requires more research.

Along with these results, it was anticipated that the expression of the other candidate genes would also be significantly impacted either by the presence of NaB or LPS. Specifically, it was expected that expression of *IL-10* would be up-regulated in the presence of NaB [27]. Also, it was hypothesized that expression of *PGRP* and *caspase-b* would be up-regulated in the presence of LPS [28,29], and *claudin-b*, *occludin-a,* and *occludin-b* would be up-regulated in the presence of NaB [30,31]. However, no significant changes in expression were observed, although we observed trends for *PGRP*, *occludin-a*, *IL-10*, *IL-1β,* and *TNF-α*. The trends observed for *PGRP*, *IL-1β,* and *TNF-alpha* were consistent with the literature [23,32,33,34], while the trends observed for *occludin-a* and *IL-10* were not [35,36,37]. We observed that *IL-1β* and *TNF-alpha* both tended to be down-regulated in the presence of NaB, which makes sense as both *IL-1β* and *TNF-alpha* are pro-inflammatory cytokines and NaB elicit anti-inflammatory effects [23,32,33].

Conversely, we observed that *IL-10* expression tended to be down-regulated in the treatment groups exposed to either 3000 μM NaB + 60 μg/mL LPS or 6000 μM NaB when compared to the group exposed to 60 μg/mL LPS alone. The results of *IL-10* were interesting in that while there was no significant difference observed in expression between the positive and negative controls, trends were observed, indicating that exposure to higher concentrations of NaB reduced expression of *IL-10* when compared to the positive LPS control. While it makes sense that *IL-10* expression would not be induced in the group exposed to 6000 μM NaB, as this treatment would not cause inflammation, we expected to see up-regulated expression of *IL-10* when exposed to both 3000 μM NaB and 60 μg/mL LPS, especially when compared to treatment groups only exposed to EW or NaB, as IL-10 is an anti-inflammatory cytokine [27]. Moreover, a study by Zheng et al. [30] observed that microbial-derived butyrate can improve epithelial barrier function in human intestinal epithelial cells (IECs) by increasing the expression of *IL-10 receptor α subunit* (*IL-10RA*), which would potentially increase responsiveness to IL-10. Zheng et al. [30] observed that *IL-10RA* transcript expression increased by 140.8 ± 39.3-fold in Caco-2 cells and 97.0 ± 21.5-fold in T84 cells, while epithelial *IL-10* was increased by 30 ± 2.5-fold at most, which suggests that *IL-10* expression induced by butyrate is limited when compared to induced *IL-10RA*. Future work is needed to clarify if an increase in IL-10RA expression can be induced by butyrate in zebrafish.

Similarly, we observed that *occludin-a* tended to be down-regulated in the presence of 6000 μM NaB when compared to the group exposed to only 60 μg/mL LPS. This also was not consistent with the literature, as occludin-a is a constituent of tight junctions, whose function NaB reportedly improves [30,31]. It is possible that this down-regulation was due to a lack of inflammatory stimulus in the treatment group exposed to only 6000 μM NaB. Another possibility is that NaB has quenched occludin-a expression in comparison to the negative control. This quenching manifests as a difference when compared to the LPS positive control, but upon the combination with LPS, NaB does not appear to show an effect. Furthermore, it was observed by Zheng et al. [30] that exposure to butyrate in human IECs down-regulated the expression of human *occludin* in Caco-2 cells and had no significant effect on the expression of human occluding in T84 cells, suggesting that down-regulation of *occludin-a* in zebrafish, homologous to human occludin, may be beneficial in terms of epithelial barrier function.

Lastly, *PGRP* tended to be upregulated in fish from the 60 μg/mL LPS positive control when compared to the negative control group. The 3000 μM NaB + 60 μg/mL LPS treatment interestingly attenuated this response, which supports the possibility that PGRP may act as an LPS receptor in zebrafish. Traditionally, the TLR4 pathway is responsible for detecting LPS, but it has been shown that the zebrafish TLR4 receptors do not bind directly to LPS [38,39]. PGRP has been proposed as a means of LPS recognition in zebrafish, as zebrafish are the only teleost species in which PGRP has been verified to bind to peptidoglycan (PGN), a pathogen-associated molecular pattern (PAMP) of Gram-positive and Gram-negative bacteria [29], and PGRP has demonstrated to be capable of recognizing and binding to common components of bacteria, including LPS [34,40]. Mammalian PGRPs have specific binding sites for the muramyl-peptide fragment of bacterial PGN, more abundantly found on the surface of Gram-positive bacteria [41], as well as additional specific binding sites for bacterial lipoteichoic acid and LPS, found on the surface of Gram-negative bacteria such as *Pseudomonas* spp. [40]. The observed increased expression of *PGRP,* especially in the presence of LPS, supports the idea that PGRP recognizes and binds to LPS in zebrafish instead of TLR4, but more work is needed to confirm this.

**Figure 4:**
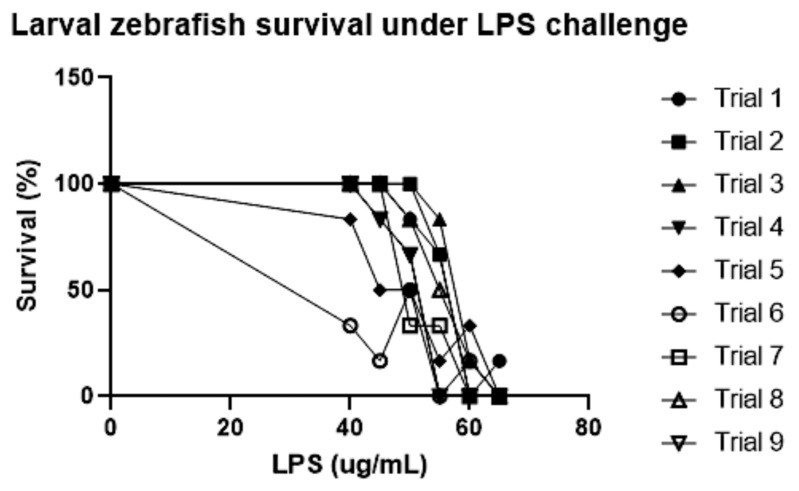
Survival of larval zebrafish (96 hpf) following 24 h exposure to *P. aeruginosa* lipopolysaccharide (LPS) (n = 6/trial). These nine trials were repeated at independent times.

Zebrafish have been known to have a high degree of genetic variation, which may contribute to variation in responses across treatments [35]. In this study, embryos were collected weekly from different pairings of adult zebrafish from the broodstock to try and normalize this variation, but it is probable that this method of variation control was not enough. Indeed, our historical LPS concentration-response survival data clearly illustrates this variation (Figure 4). To help overcome this, independent replicate experiments need to be performed, as we have done, and expected survival endpoints need to be simultaneously confirmed while investigating gene expression. Despite doing this, we still found relatively few significant changes in the expression of candidate genes. Since whole-body tissue was used to assess changes in gene expression, and we required 50 fish per treatment to obtain enough material for total RNA isolation, it is likely that more subtle organ or tissue changes in gene expression were masked using this whole-body tissue approach. Future studies may want to carry out the isolation of macrophages to carry out single-cell gene expression analysis or repeat such experiments using adult fish and in-feed NaB to assess organ- and tissue- changes in gene expression.

## 4. Conclusions

Taken together, the results from this study confirm the protective attributes of NaB during a subsequent immune challenge, with exposure to 6000 μM NaB significantly increasing survival rate following treatment with 60 μg/mL LPS. This study also demonstrated that 6000 μM NaB exposure significantly down-regulated the expression of *TNF-α*, consistent with findings in the literature. Further investigation with more variation control measures is needed to better assess and confirm the effects of both NaB and LPS on the expression of the other candidate genes in this study.

## 5. Materials and Methods

### 5.1. Zebrafish Husbandry

Zebrafish larvae were obtained from the Hagen Aqualab (Guelph, ON, Canada) by breeding adult zebrafish from broodstock of wild type and green fluorescent protein-tagged genetically modified fish. Adult zebrafish were housed in 5 L tanks at a stocking density of 5–8 fish/L with a photoperiod setting of 14 h light and 10 h dark. The recirculation system was supplied with deionized water to maintain water quality, and sponge filters were cleaned weekly. The water was analyzed weekly as follows: temperature at 28 °C, dissolved oxygen at ~90% saturation, pH at 7.0–7.5, conductivity at 300 μS, ammonia at 0–2 ppm, and nitrite at 0–1 ppm.

To breed the zebrafish, randomly selected parents were collected from their respective tanks using a net placed into a breeding tank containing approximately 1 L of water with an egg separator, at a ratio of two males to three females, which were separated by sex overnight using a divider. The next morning, at approximately 9:00 AM, the adult fish were relocated into a new tank containing a low level of fresh water with the divider removed, and the tank was tilted on an angle to imitate a shoal. After 3 h, the fish were returned to their original rearing tanks, and the eggs from the mating tank were collected into commercial egg water (EW; 60 mg/L Instant Ocean Sea salts. Blacksburg, VA, USA) containing 3 ppm methylene blue to be taken to the lab; the addition of methylene blue water helped to distinguish and remove dead eggs, and these dead eggs were removed upon arrival at the lab. The remaining viable eggs were rinsed twice with EW and kept in petri dishes stored at 28.5 °C (~150 eggs/dish) in an incubator until 72 h post-fertilization (hpf).

### 5.2. Preparation and Determination of Nab and Lps Concentration

Both NaB and *P. aeruginosa* LPS were purchased from Sigma-Aldrich. Stock solutions of 100 mg/mL NaB (Sigma-Aldrich) and 1 mg/mL LPS (Sigma-Aldrich) were prepared using EW, and these were frozen as 100 μL aliquots at −20 °C until needed. Exposure concentrations were prepared by diluting these stock solutions with more EW. NaB was diluted to concentrations of 500, 3000, and 6000 μM, which is equivalent to 55, 330, and 661 μg/mL, respectively, while LPS was diluted to concentrations of 55, 57.5, and 60 μg/mL. In a preliminary trial conducted in our lab, potential NaB toxicity was assessed to ensure no detrimental effects due to NaB; 7 concentrations were assessed (10, 50, 100, 500, 3000, and 6000 μM), plus an EW negative control. Results indicated that NaB had no significant toxic effects on zebrafish larvae during the 24 h exposure period (data not shown), and the levels 500, 3000, and 6000 μM were chosen after reviewing the zebrafish literature of similar experiments [19].

Lethal LPS concentrations were determined based on historical LPS concentration responses conducted by our laboratory (Figure 4); three LPS treatment levels were chosen (55, 57.5, or 60 μg/mL) based on the narrow range of lethal toxicity, and 60 μg/mL LPS was chosen to assess changes in gene expression.

### 5.3. Larval Zebrafish NaB Exposure and Lethal LPS Immune Challenge

At 72 hpf, the petri dish that housed the zebrafish eggs was removed from the 28.5 °C incubator, and 96 hatched larvae were transferred into a 96-well plate by pipetting one fish per well containing 200 μL of EW.

The NaB exposure solutions (500, 3000, and 6000 μM) were prepared, and 200 μL was used per well to replace the EW containing each larva. Following the introduction of NaB, the 96-well plate was returned to the 28.5 °C incubator for 24 h. At 96 hpf, the larvae were examined for survival using an inverted microscope based on heartbeat, and 99% viability was observed regardless of concentration.

The 96-well plate was further divided into four main groups: a control group receiving EW and the three LPS challenge groups. The wells for the control group had their previous NaB solutions replaced with EW, while the wells for the LPS challenge groups had their NaB solutions switched out for LPS at concentrations of 55, 57.5, or 60 μg/mL. The sample sizes in the trials varied from 6 to 12 fish/treatment/challenge (n = 6–12), depending on how many plates were used each week. After the wells had been filled with 200 μL of the new respective solutions for the LPS immune challenge, the plate was returned to the 28.5 ℃ incubator and left overnight. Survival was again assessed at 120 hpf under a microscope, and any remaining live fish were euthanized with 0.1% clove oil (Sigma, St. Louis, MO, USA) and disposed of accordingly. This experiment was repeated independently nine times: three times without and six times with parallel mRNA collection. The initial three trials were comprised of 4 NaB treatments (0, 500, 3000, and 6000 μM) and four LPS treatments (0, 55, 57.5, and 60 μg/mL), while the remaining six trials run in parallel with the mRNA collection included 4 NaB treatments (0, 500, 3000 and 6000 μM) for the bioassay and two LPS treatments (0 and 60 μg/mL) for mRNA extraction.

### 5.4. Zebrafish Collection and RNA Extraction

For this gene expression study, visualization of the experimental timeline can be found in Figure 1. To extract enough mRNA to analyze, it was determined that a minimum of 50 larvae per exposure concentration would be needed to obtain sufficient total RNA. A 24-well plate was used, with each well representing an exposure group containing 3 mL of solution and ~50 fish/well. Fish were sacrificed at 6 h post-LPS challenge (102 hpf) since this was found to be the last time point without accumulation of LPS-induced mortalities. The zebrafish larvae from each exposure group were lysed using a mini blender in Trizol, and total mRNA was extracted using an RNeasy extraction kit (QIAGEN, Germantown, MD, USA). The quantity and purity (A260/A280) of isolated RNA samples in the present study were determined using an Agilent BioTek Take3 microvolume plate reader (Cytation5, Agilent Technologies, VT, USA). RNA samples with an A260/A280 ratio between 1.9 and 2.1 were considered suitable for further qPCR analysis. The samples were kept on ice throughout the extraction procedure and were immediately transferred to the −80 °C freezer for storage until they were used for cDNA synthesis and qPCR analysis.

### 5.5. Gene Expression (qPCR)

The purified RNA (5000 ng) was reverse transcribed to cDNA using the High-Capacity cDNA Reverse Transcription Kit (Applied Biosystems, USA). Genes for qPCR analysis were chosen by reviewing literature based on known and potential effects of NaB. Once a list of genes was selected, primer sequences were identified from the zebrafish literature [36,37,42,43,44,45] The list of genes chosen for this experiment included *β-actin*, *18S ribosomal RNA* (*18S rRNA*), *tumor necrosis factor α* (*TNF-α*), *peptidoglycan recognition protein* (*PGRP*), *myeloid differentiation primary response 88* (*MyD88*), *interleukin 1β* (*IL-1β*), *suppressor of cytokine signaling* (*SOCS1*), *IL-10*, *claudin-b*, *occludin-a*, *occludin-b* and *caspase b*. *β-actin* and *18S rRNA* were the housekeeping genes and are commonly used as housekeeping genes across species [46]. However, since *18S rRNA* was found to be the most stable by assessing its consistent expression in all samples, it was selected for the final normalization during qPCR analysis. TNF-alpha and IL-1β are pro-inflammatory cytokines that are released in response to bacterial stressors and their cellular components, including LPS [47]. SOCS1 and IL-10 are cytokines whose expression is induced by the presence of pro-inflammatory cytokines such as TNF-alpha and IL-1β, and they respond by regulating the immune system, for example, by resolving inflammatory cascades [48]. MyD88 is a common adaptor protein in the downstream signaling pathways of many mammalian toll-like receptors (TLRs), including TLR4, which is responsible for recognizing and binding to LPS and inducing downstream signaling events in mammals [49]. PGRP and caspase b also both recognize LPS, and caspase b is responsible for inducing noncanonical inflammasome activation [28,34]. Lastly, claudin-b, occludin-a, and occludin-b are tight junction proteins responsible for regulating epithelial cell permeability in larval zebrafish [43,50]. A total of 13 primer sets were synthesized at the Laboratory Services Division (Guelph, ON, Canada). Primers and their sequences can be found in Table 1.

After PCR conditions were optimized and PCR product size validated by gel electrophoresis, the relative quantitative expression of target genes was determined using the StepOnePlus™ Real-Time PCR System (Applied Biosystems, Burlington, ON, Canada). Real-time PCR was performed using SYBR^®^ Green qPCR supermix, and the PCR conditions consisted of 50 °C for 2 min, 95 °C for 2 min, 40 cycles of 95 °C for 15 s, 60 °C or an optimized annealing temperature for 30 s, and 72 °C for 30 s. Each qPCR plate contained two non-template controls (NTC) and five standards, which were created using a mix of the samples and serially diluted, along with individual samples. Dissociation curves were generated at the end of amplification to ensure the presence of single amplified products of appropriate size. The cycle threshold (Ct) values for each sample were obtained by StepOnePlus™ software v2.3.

### 5.6. Statistical Analysis

Survival data from the LPS challenge were presented as a binary response, ‘1’ indicating survival and ‘0’ indicating death, while data from qPCR analysis were presented as a fold-change relative to the *18S rRNA* gene, as *18S rRNA* was found to have the most stable expression across all samples. To quantify gene expression. Both sets of data were analyzed using SAS^®^ OnDemand for Academics. Normality was assessed for both sets of data by assessing the distribution of residuals and by using the Shapiro–Wilk test, and the results indicated that the survival data were not normally distributed, while the qPCR data were normally distributed. Therefore, a nonparametric Kruskal–Wallis analysis (post-hoc) was chosen for the survival data, conducted with SAS using the NPAR1WAY procedure, and pairwise comparisons between all treatments were performed using a Dwass, Steel, Critchlow–Fligner multiple comparisons test. For the qPCR data, an ANOVA was conducted on SAS using the MIXED procedure for each candidate gene, and pairwise comparisons between each treatment against the respective control or LPS treatment group were performed using a Dunnett *t*-test. Data are presented as fold-change representing gene expression, and significance was determined as *p* < 0.05, and trends were noted at 0.1 < *p* < 0.05.

## Figures and Tables

**Table 1:** List of immune-related zebrafish candidate genes and their sequences.

Function	Gene		Primer(s)	Annealing Temperature (°C)	Accession Number
Housekeeping	*β-actin* [22]	forward	CGAGCAGGAGATGGGAACC	61.8	NM_131031
		reverse	CAACGGAAACGCTCATTGC		
	*18S rRNA* [23]	forward	TCGCTAGTTGGCATCGTTTATG	60	BX296557.35
		reverse	CGGAGGTTCGAAGACGATCA		
Immune function	*MyD88* [20]	forward	TCCACAGGGACTGACACCTGAGA	61.8	NM_212814
		reverse	GCTGAGTCTTCAGCACAGCAGAT		
	*SOCS1* [25]	forward	TGGAAGCGGCGACGAGAGTT	60	NM_001003467
		reverse	CGGCTTGAAATGTGTCTGG		
	*IL-1β* [22]	forward	ATGCTCATGGCGAACGTC	61.8	NM_212844
		reverse	TGGTTTTAGTGTAAGACGGCACT		
	*TNF-alpha* [22]	forward	AGGCAATTTCACTTCCAAGG	60	NM_212859
		reverse	AGGTCTTTGATTCAGAGTTGTATCC		
	*IL-10* [22]	forward	AAGCGGGATATGGTGAAATG	60	NM_001020785
		reverse	CCCCCTTTTCCTTCATCTTT		
Tight junction function	*claudin-b* [23]	forward	AGACAGCGGAAAATACACAGC	60	NM_131763
		reverse	TGAGCCTCAATGTCCAACAA		
	*occludin-a* [23]	forward	GGGTCTGCTGGCTGACTATC	61.8	NM_212832
		reverse	GAATCTCCACGGGACTTTCA		
	*occludin-b* [23]	forward	GACCATTAAGGATGGCCTCA	60	XM_005171844
		reverse	GCTGAGCAGCACTGACTTTG		
LPS recognition	*Caspase b* [24]	forward	ATGGAGGATATTACCCAG	63	NM_152884
		reverse	TCACAGTCCAGGAAAC		
	*PGRP* [21]	forward	TGGACAATCGAACATGGGACGA	60	MGC04209
		reverse	CCGTACGTATGTGCCCCGAC		

Notes: *18S rRNA*, 18S ribosomal RNA; *MyD88*, Myeloid differentiation primary response 88; *SOCS1*, Suppressor of cytokine signalling 1; *IL-1β*, Interleukin 1β; *TNF-alpha*, Tumor necrosis factor α; *IL-10*, Interleukin 10; *PGRP*, Peptidoglycan recognition protein. Sources of primers are present as superscript reference numbers.

## Data Availability

The datasets used and analyzed during the current study are available from the corresponding author upon request.
